# Evolutionary Genetics of Cacti: Research Biases, Advances and Prospects

**DOI:** 10.3390/genes13030452

**Published:** 2022-03-01

**Authors:** Fernando Faria Franco, Danilo Trabuco Amaral, Isabel A. S. Bonatelli, Monique Romeiro-Brito, Milena Cardoso Telhe, Evandro Marsola Moraes

**Affiliations:** 1 Departamento de Biologia, Centro de Ciências Humanas e Biológicas, Universidade Federal de São Carlos (UFSCar), Sorocaba 18052-780, Brazil; franco@ufscar.br (F.F.F.); danilo.trabuco@gmail.com (D.T.A.); romeiro.monique@gmail.com (M.R.-B.); milena.telhe@gmail.com (M.C.T.); 2 Programa de Pós-graduação em Biologia Comparada, Faculdade de Filosofia, Ciências e Letras de Ribeirão Preto, Universidade de São Paulo (USP), Ribeirão Preto 14040-901, Brazil; 3 Departamento de Ecologia e Biologia Evolutiva, Universidade Federal de São Paulo (UNIFESP), Diadema, São Paulo 04021-001, Brazil; belbonatelli@gmail.com

**Keywords:** Cactaceae, literature survey, evolutionary genetics, genomics

## Abstract

Here, we present a review of the studies of evolutionary genetics (phylogenetics, population genetics, and phylogeography) using genetic data as well as genome scale assemblies in Cactaceae (Caryophyllales, Angiosperms), a major lineage of succulent plants with astonishing diversity on the American continent. To this end, we performed a literature survey (1992–2021) to obtain detailed information regarding key aspects of studies investigating cactus evolution. Specifically, we summarize the advances in the following aspects: molecular markers, species delimitation, phylogenetics, hybridization, biogeography, and genome assemblies. In brief, we observed substantial growth in the studies conducted with molecular markers in the past two decades. However, we found biases in taxonomic/geographic sampling and the use of traditional markers and statistical approaches. We discuss some methodological and social challenges for engaging the cactus community in genomic research. We also stressed the importance of integrative approaches, coalescent methods, and international collaboration to advance the understanding of cactus evolution.

## 1. Introduction

Cactaceae (Caryophyllales, Angiosperms) is the major lineage of succulent plants, originating during the Eocene-Oligocene transition [1,2,3], and it is recognized by its remarkable diversity [4,5]. It is one of the most conspicuous examples of species radiation in the Americas, with intense diversification in the last 10 million years [1]. Cactus species have been traditionally studied by morphologists, and several taxonomic reorganizations have been proposed in the last century (e.g., [4,6,7,8]). As an emblematic and ecologically relevant group, many evolutionary studies using genetic-based markers have been published in recent decades (e.g., [9,10,11,12,13,14,15]). However, in several examples, classical molecular markers often lack sufficient information to resolve phylogenetic relationships (e.g., [16,17,18,19]) and genetic variation at intraspecific level (e.g., [20,21,22]). Considering the efforts undertaken so far in sampling traditional molecular markers for the family Cactaceae, and that the increasing accessibility to new sequencing technologies [23,24] has fostered genomic sampling in Cactaceae, we believe that a turning point has been reached and that this is the appropriate context for a review of current knowledge achieved using those molecular markers.

Evolutionary studies using molecular markers have been routinely performed to address questions on the natural history of living organisms, such as life histories, population genetics, gene flow, taxonomy, and evolutionary relationships [25]. Moreover, target taxa may be useful as a biological model to study broader aspects of their habitats and their geography, such as the environmental fluctuations and evolution [26]. In this regard, cacti have relevant idiosyncrasies to be considered biological models for evolutionary studies. For example, given that it is a recent radiated group [1], many clades might be useful to study recent and explosive diversification. Moreover, as many species may hybridize in nature (e.g., [13,27,28]), cacti may provide many insights into hybrid zones, species cohesion, and species delimitation. Finally, due to its intrinsic association with drought stress, cacti are useful models for biogeographic approaches aiming to investigate the effects of Quaternary climate oscillations on xeric biomes (e.g., [21,29,30,31,32]). As a result, many studies with an evolutionary focus have been conducted on cacti in recent decades.

Here, we performed a literature survey to obtain detailed information and summarize important aspects of molecular marker-based studies investigating cactus evolution. Specifically, we address historical tendencies and the combined use of molecular markers, phylogenetics, biogeography, species delimitation, hybridization, and genome assemblies. Many advances, limitations, and biases in cactus research are presented. We emphasize the importance of an integrative approach in the future to properly understand family evolution and alternative methods for data analysis. Finally, we discuss the methodological challenges involved in genomic research as well as the need to integrate the cactologist community, especially those from low-income countries with high cactus diversity.

## 2. Materials and Methods

The core of this review focused on evolutionary genetics works, mostly related to biodiversity descriptions, such as phylogenetic, phylogeography, population genetics, species delimitation, and hybridization studies. For this purpose, studies of introduced/nonnative greenhouse cacti and those that did not use molecular markers were not considered. The literature survey was carried out in the Web of Science ISI database (til 31 December 2021) using the following terms: “phylogeograph*” + “Cact*”; “phylogenet*” + “Cact*”; “delimit*”+ “Cact*”; “hybridization” + “Cact*”. Specific searches for microsatellite markers and their transferability were applied using the terms “SSR” + “transferability” + “Cact*” and “microsatellite” + “transferability” + “Cact*”. This non-exhaustive survey was followed by the inspection of the papers’ titles, abstracts, and keywords. Based on our interpretation of the main authors’ aims, we classified the papers into three main topics: phylogeny, population genetics, and phylogeography. From each paper, we recorded the following information, partitioned in (i.) publication year, DOI, first/last author country; (ii.) taxonomy (taxon/clade), species geographic occurrence (based on dominion proposed by [33]); (iii.) molecular marker type; (iv.) main topics; (v.) methodological approach; (vi.) taxon-specific goals; (vii.) biogeographic hypothesis(es); and (viii.) main conclusions (detailed in Tables S1 and S2 in the Supplementary Information).

An additional survey (til 31 December 2021) was performed to recover studies applying functional genomic approaches into the study of Cactaceae (genome and transcriptome datasets). For that, we used the following terms: “genom*” + “Cact*”, “Transcriptom*” + “Cact*”, and “RNA-Seq” + “Cact*”. We checked the papers’ titles, abstracts, and keywords as mentioned above (Table S3). The recorded information was as follows: (i.) publication year, DOI, first/last author country; (ii.) taxonomy (taxon/clade); (iii.) genomic source (nuclear, plastid, or mitochondrial); and (iv.) main conclusions (detailed in Table S3 in the Supplementary Information). We generated two flow diagrams based on Page et al. [34] to explore our survey method (Figure S1).

## 3. Results

**Figure 1:**
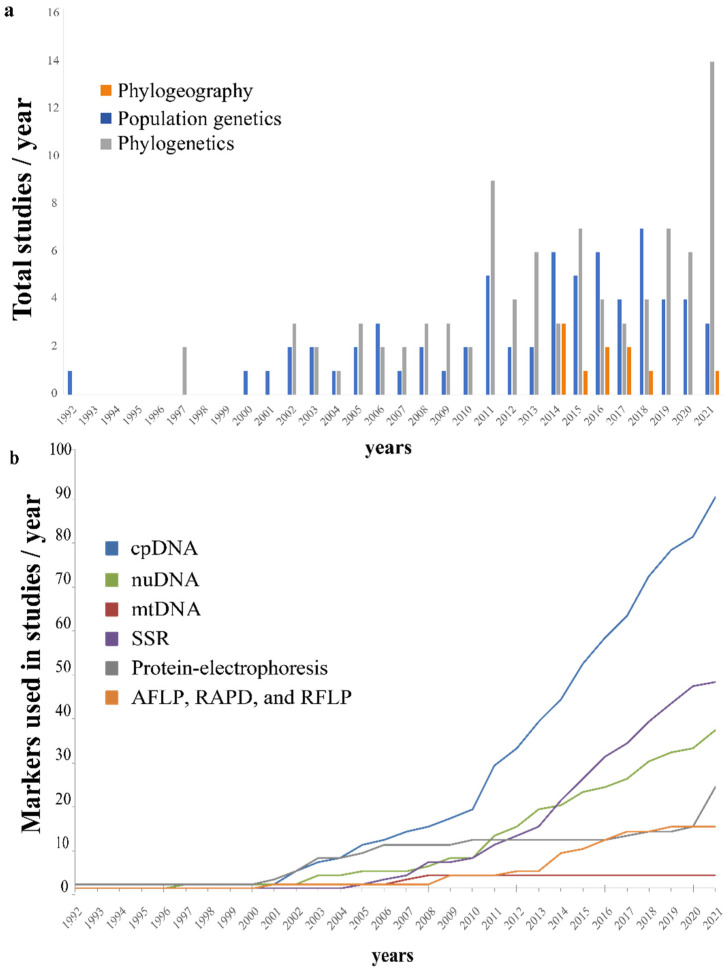
The number of evolutionary studies in Cactaceae, tabulated by main topics described in the text (**a**), and cumulative curve of major molecular markers used in the studies (**b**), conditioned on survey data spanning 1992–2021.

**Figure 2:**
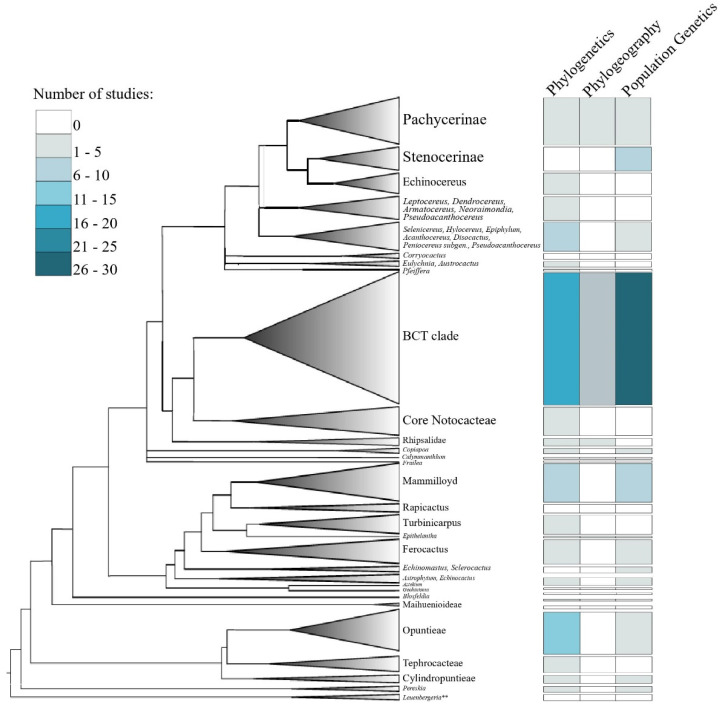
Distribution of evolutionary studies on the cactus backbone phylogeny (Adapted from [19] permission has been obtained).

**Figure 3:**
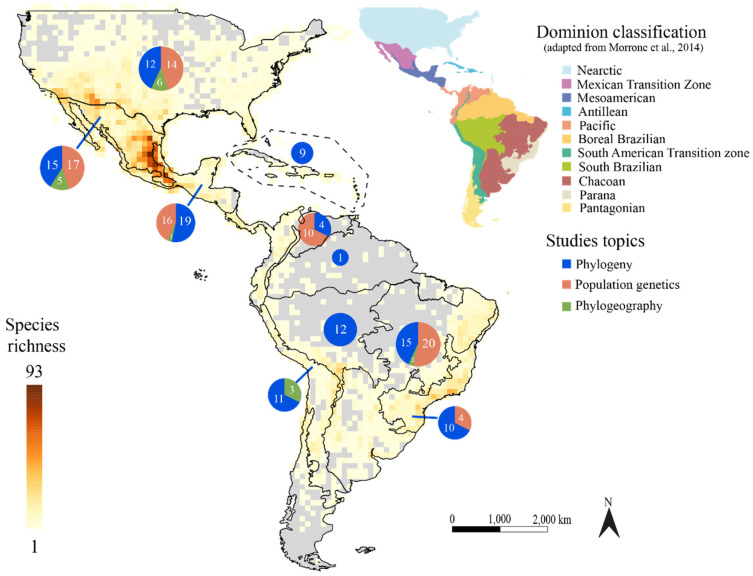
Distribution of species richness within the Cactaceae family according to the dominion classification of Morrone et al. [33]. The number of studies in the main regions is shown in blue circles.

We retrieved 181 articles published from 1992 to 2021, of which 166 papers were mainly associated with phylogeny, population genetics, and phylogeography topics (Tables S1 and S2), while 27 papers were published since 2015 and were related to genomic and transcriptome assemblies (Table S3). Ninety papers were categorized in the phylogeny, 67 in the population genetics, and nine in the phylogeography topics (Tables S1 and S2). Most of these papers were led by researchers from the United States, Mexico, and Brazil (Figure S2). The number of papers in these fields has grown in the past two decades (Figure 1a). As expected, PCR-based markers are historically prevalent in evolutionary studies of cacti, but the number of studies using genome-wide markers has been constantly growing in the last five years (Figure 1b). Most studies are concentrated in two cactus clades: BCT (Browningieae-Cereae-Trichocereae) and Opuntiae (Figure 2). The Nearctic, the Mexican Transition zone, the Mesoamerican, and the Chacoan Domains (sensu [33]) are the most represented biogeographic domains in our literature survey. However, these studies covered only a small fraction of all the species richness and endemism of the Neotropical region (Figure 3).

**Figure 4:**
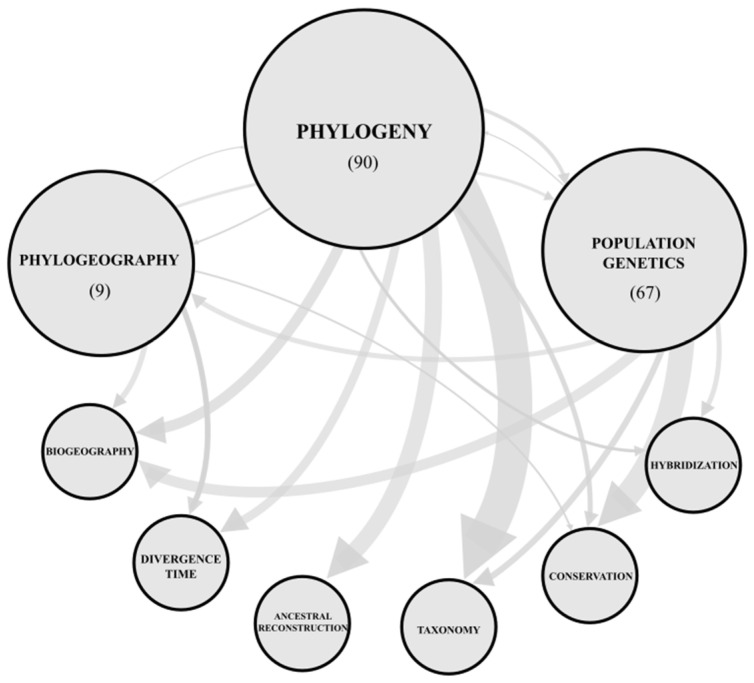
Network showing connections among the main topics addressed in evolutionary studies of Cactaceae. Circle sizes are proportional to the number of studies in each category (see Table S1), and line thickness is proportional to the number of connections between categories.

The phylogenetic category is the most multidisciplinary topic, connected to the other two topics (i.e., population genetics and phylogeography), and with all approaches identified in the recorded papers (Figure 4). Many phylogenetic studies have been conducted to answer questions on divergence time, ancestral reconstruction, taxonomy, and species circumscription. Conversely, phylogeography and population genetic categories are less associated among them, and among study approaches. The population genetics’ studies were most interested in conservation purposes, although they also displayed fewer interactions with other topics.

Many of the recovered papers (>50%) relating to genomic and transcriptome assemblies were published in the past two years, most of which were related to plastome or transcriptome assembly reports (Table S3). This suggests a growing interest in cactus genomics as sequencing technologies become more feasible to researchers.

## 4. Discussion

### 4.1. Molecular Markers

The use of molecular markers for phylogenetic, population genetics, and phylogeographic purposes has followed historical tendencies [35]. Protein electrophoresis (allozymes and isozymes), restriction fragment length polymorphism (RFLP), random amplified polymorphic DNA (RAPD), and amplified fragment length polymorphism (AFLP) were the primary markers used in evolutionary studies in cacti (26 studies), but they have lost their importance in the past decade. Since the 2000s, there has been a transition to more polymorphic PCR-based markers (Figure 1b). As a result, most of the studies started to use plastid DNA sequences (cpDNA; 90 studies), followed by Simple-sequence repeats (SSRs; 48 studies) and nuclear DNA (nrDNA; 37 studies) markers (Figure S3). The use of high-throughput genomic data has been applied in the past decade, comprising 10 studies.

Approximately 28% of the studies (45 studies) combined the use of multiple markers. Most of them employed cpDNA plus nrDNA (31 studies), followed by the combination of cpDNA plus SSR markers (seven studies) (Figure S3). The mtDNA (mitochondrial DNA) marker was used in only three phylogenetic studies, probably due to the slower evolution, unstable structure, and higher recombination rates of this genome in plants [36,37]. On the other hand, plastid markers have been traditionally used for evolutionary studies within Cactaceae, favored by the availability of universal primers to segments potentially informative for multiple phylogenetic scales across angiosperms [38]. Although plastid regions presented highly informative regions to resolve phylogenetic relationships within Cactaceae tribes (e.g., [39]), these regions generally showed low potential to resolve deep internodes at the species level (e.g., [18,40]). Furthermore, plastid markers are prone to lineage-specific variations even among closely related species [41]. For cacti, this issue was tested for the genus *Cereus*, agreeing with the non-correspondence between the variation in plastid regions at the inter- and intraspecific levels [22,42]. The practical consequence is the need for a preliminary essay for screening molecular variation in candidate regions to be applied in the target group, a step that may be laborious. Nonetheless, it is important to highlight the valuable phylogenetic information enclosed in the whole plastome data. In Cactaceae, the use of a complete chloroplast genome has been recently explored [43,44], highlighting the potential of plastomes for phylogenetic purposes in this family.

A large proportion of studies using PCR-based technologies combine markers from plastid and nuclear genomes (Figure S3). Although the ribosomal internal transcribed region (ITS) presents extensive paralogy due to the absence of concerted evolution in some cactus clades, such as *Mammilaria* [45,46], it was one of the most common nrDNA marker adopted in combined studies, in addition to the phytochrome C gene (*PhyC*). The *trnK*, *matK*, *trnL-F*, and *trnL* were the most used cpDNA markers. The combination likely occurred due to a low mutation rate of cpDNA markers compared to nrDNA regions [36,47] and low sequence variation of cpDNA among closely related species or among conspecific populations (e.g., [21,48,49]). Even so, the use of multiple independent molecular markers has been a successful strategy to resolve phylogenetic relationships within rapid and recent divergent groups of Cactaceae [21,50,51,52]. Furthermore, the integrative use of plastid and nuclear genome information allows us to understand many biological issues in plants, such as patterns of plastid-nuclear discordance caused by differential dispersal between pollen and seeds.

The SSR is the second most commonly used type of molecular marker (Figure 1b). Microsatellites show recognized advantages to population genetics’ studies [53,54]. Moreover, the possibility of effectively transferring these markers across taxa may overcome the challenges, cost, and time-consuming development of new SSR primers [53,55]. In cacti, this technique has been applied with success (Table S1) at both the cross-species and cross-genera levels [28,56,57,58,59,60]. In addition to the possibility of cross-amplification using heterologous primers, the emergence of NGS technologies has allowed the description of new SSR loci from bioinformatic surveys (e.g., [51,61]). In this context, SSRs remain a marker of choice for exploring intraspecific genetic variation in cactus studies, even in the genomic era.

Although relatively few genomic-scale studies were recovered in our literature survey, we noticed an increasing use of genome-wide markers in the past five years (Figure 1b). For example, different methods to sequence a reduced representation of genomes have been used in evolutionary studies on cacti, resolving rapid and recent diversification in this group (e.g., [50,62,63]). In addition, de novo assembled genomes, transcriptomes, and plastomes have been successfully used in some cacti groups (e.g., [32]). These recently available genomics data are a valuable source to assist target enrichment sequencing approaches, enabling the identification of hundreds of orthologous regions (e.g., [64]). This technique may enable obtaining sequences from multiple and unlinked nuclear orthologous genes to perform evolutionary studies at multiple phylogenetic scales on this group.

Representative sampling is a critical factor when using molecular markers for intraspecific inferences. For instance, the sample size is an outstanding predictor of population structure inference [65], evidencing a bias caused by inadequate sampling on within- and among-population variation. In another way, when thousands of loci are sampled by high-throughput sequencing, very small sample sizes (i.e., two individuals) seem to be enough to properly sample within-population variation [66]. Cactaceae diversity has several attributes that demand more sampling efforts than less diverse plant groups. For example, despite the small range size of many cacti species, which would demand lower sample sizes, the extreme genetic differentiation observed even among close but physically isolated populations (e.g., [48,67]) requires a spatially more accurate sampling of the populations. Ultimately, both the robustness and accuracy of phylogenetic and population genetic inferences depend on the appropriate sampling of the genetic diversity within the taxon.

### 4.2. Phylogenies

The phylogenetic studies on Cactaceae are concentrated on two main clades, Browningeae-Cereae-Trichocereae (BCT) and Opuntiae (Figure 2; Table S2). These clades are among the most diverse groups in this family (Figure 2) and are mainly located in two centers of the highest cacti diversity (Figure 3; [19]). However, some clades are poorly explored in a phylogenetic context, such as the subtribes Stenocereinae and *Corryocactinae,* in addition to the *incertae sedis* genera *Calymmanthium* and *Frailea*. The poor representation of these clades in phylogenetic studies might be because most of the diversity in these taxa occurs outside of countries where cactus phylogenetics studies are concentrated (USA, Mexico, and Brazil; Figure S1). In the same way, some hotspots of cacti diversity, such as the Andes/Chaco region, are rarely represented in cacti phylogenetic studies, probably due to the low number of researchers interested in the molecular phylogeny of cacti in the countries within these regions.

In Cactaceae, poor resolution and the presence of recalcitrant nodes are common observations [19]. The recent and rapid diversification of major clades within this family [1,2] coupled with the use of few molecular markers are likely behind the lack of phylogenetic resolution and node support in phylogenies encompassing different taxonomic levels. Single- or few-locus phylogenetic approaches provide insufficient genetic information to overcome the phylogenetic bias produced by natural phenomena in the genome of these taxa, such as incomplete lineage sorting (ILS), hemiplasy, and hybridization events [15,68]. An alternative approach to overcome these challenges is to use multiple loci data under coalescent phylogenetic methods, increasing the chance of obtaining more robust and accurate phylogenies [69,70]. Indeed, this is a rising trend observed in evolutionary studies within Cactaceae (see Molecular Markers topic). It is worth noting that the rapid differentiation experienced by many cacti lineages [1] may lead to short-deep internodes characteristics of “anomaly zones” in the tree [71], imposing additional challenges to phylogenetic reconstruction, such as those observed in the *Cereus*-*Cipocereus* branch [62]. Many cactus phylogeneticists will need to deal with these theoretical/methodological issues, regardless of the amount of dataset used.

The majority of recovered phylogenetic studies in Cactaceae implement the concatenation approach. By equalizing conflicting genealogical histories of independent genes in a super matrix, this approach may produce well-supported misleading phylogenetic relationships [72]. A way to overcome this challenge is to use methods that incorporate the different coalescent time and genealogical history of each locus independently (e.g., ASTRAL-III [73]; SVDquartets [74]; STARBEAST2 [75]; SNAPP [76]). Therefore, the use of coalescent phylogenetic methods is extremely important to be applied in groups that lead to intensive gene tree discordance, as occurs in cacti [15].

### 4.3. Biogeography

Biogeographic studies using molecular markers in Cactaceae have become an area of interest only since the beginning of the 2000s (Table S1). In the last two decades, we observed 33 studies that applied biogeographic reconstruction methods within the three main topics proposed here; approximately 50% are based on phylogenetic inference within genera or higher taxonomic levels (16 studies; Figure 4). Downstream analyses related to phylogenetic approaches, such as divergence time estimates, are mandatory for well-calibrated biogeography reconstruction [77]. The association between the reconstruction and the test of postulated biogeographic hypotheses is focused on understanding the patterns and processes that drive species diversification [78,79]. However, even using biogeographic/phylogeographic approaches, some of the papers do not frame their questions as particular or general biogeographical hypotheses (Tables S1 and S2) to be tested, restricting their findings to a description of ancestral geographic ranges of the target clade. It is desirable that further biogeographic studies should be more explicit regarding the biogeographic conjecture target of testing, to improve the comparisons and to identify drivers of diversification into the family.

Based on the recovered papers, we were able to identify four main associated biogeographic theories: (i.) “Geomorphology”; (ii.) “Riverine”; (iii.) “Quaternary climatic changes”; and (iv.) “Neogene aridification” (Table S4). Most of the studies suggested “Quaternary climatic changes” (ca. 72%) and/or “Geomorphology” (ca. 27%) as the most relevant hypotheses to explain range shifts, distributions, and diversification of cactus species in the Neotropics. For instance, the climatic oscillation involving the glacial cycles of the Pleistocene is thought to have promoted the expansion and contraction (fragmentation) range of populations from distinct genera, such as *Pilosocereus*, *Cephalocereus*, *Rhipsalis*, and *Echinopsis* [21,29,31,50,80]. Moreover, paleoclimates drove marine transgressions/regressions, which have been inferred to have impacted the population dynamics of coastal and island populations [3,60,81]. Geomorphologic events, such as the uplift of the Andes, Trans Mexican Volcanic Belt structures, and geomorphological compartmentalization of the Brazilian Central Plateau, also influenced the distribution and expansion of cactus species [16,21,81,82,83]. The “Neogene aridification” theory advocates the expansion and recent radiation of the Cactaceae in the last 35 Ma (Miocene to Pliocene) [1,2,13,32,84]. The “Riverine” theory was recovered only once, associated with the genus *Pilosocereus* and the eastern Brazilian rivers, such as the Sao Francisco and Doce rivers [85]. One important consideration is that alternative biogeographic hypotheses should not be considered as mutually exclusive, as most biogeographic patterns can be explained by the combined and continuous effects of different historical events [86]. Most studies accessed here show a continuous diversification trend in cactus clades from the Neogene to the Pleistocene (Table S4 and Figure S4).

The study’s topics in Cactaceae are restricted to a few biogeographic domains (Table S2), such as the Nearctic, Mexican Transition Zone, Mesoamerican, and the Chacoan sensu [33], which are still far from covering all the species richness and endemism of the Neotropics (Figure 3). This bias is intensified by a knowledge trend: political boundaries are barriers for researchers to explore large-scale diversity patterns, as most researchers concentrate their works within the limits of their countries. An additional challenge in both empirical and theoretical biogeographic studies relate to the definition of geographic operational units, which are generally set as the biomes in the study region. The delineation of biogeographic units can become quite a challenging task given the high diversity and complexity of Neotropical biomes, for example, those exhibiting a mosaic-like distribution [87,88]. Therefore, to acknowledge the limits and definitions of a biome, several and distinct particularities must be considered, such as the distribution, potential natural vegetation, and general abiotic environmental variables [89,90,91].

A biogeographical synthesis of Cactaceae is lacking as well as a more complete knowledge of diversification drivers in drylands [92]. Xeric landscapes display a complex evolutionary history, including several hotspots of diversity and centers of diversification [1,93,94]. Drylands also display great functional diversity and morphological/physiological adaptation, distinct interactions between plants (i.e., mutualism), plants plus soil (i.e., inorganic compounds), and plants plus microbiota (i.e., nematodes, bacteria, insects, and viruses [92,95,96]). Why do we have so much species richness in environments with such low water availability and nutrient-poor soil? The biogeographical theory recovered in these studies, as we cited, brought mechanisms to determine and explain part of this question. However, many other mechanisms still need to be evaluated and tested, exploring new theories, distinct cactus taxa, and new methodological and statistical approaches.

Like many other areas of biodiversity sciences, biogeography requires efforts toward an integrative approach by using organismal traits, environmental variables, and genetic data [97,98,99]. Recent studies within the field of phylogeography, for instance, have developed predictive frameworks to aggregate this information and predict patterns of diversity along with species distribution [100,101]. These approaches have taken advantage of the predictive power of machine learning and the availability of distributional, climate, and trait data housed in public databases such as Global Biodiversity Information Facility GBIF [102], Worldclim [103], and TRY [104]. Considering the importance of life-history traits to predict patterns of diversity and distribution within taxonomic groups, we argue that the development of trait-based approaches, which have already started in the phylogeography of other taxonomic groups [97,98,99,105,106], could be extended to broad applications in the biogeography of Cactaceae.

### 4.4. Species Delimitation

There are some reasons to use molecular data to assist species recognition in cacti. First, cacti experience a diverse range of threats [93,94], and molecular data may contribute to revealing unprotected cryptic diversity or identifying smuggled living cactus. Second, the family has an astonishing diversity of species and growing forms [4], in addition to a substantial interpopulation variation in morphology, which may cause taxonomic uncertainty. Altogether, these factors contribute to the origin of many “species complexes” in Cactaceae, which are systems that may impose major challenges for species delimitation [26]. Consistently, molecular markers have been used to assist species recognition and species circumscription in the cactus family (Figure 4), most of which are biased to PCR-based markers within disparate phylogenetics and population genetics approaches. However, incorporating modern sequencing technologies using coalescent-based methods is of great promise for this purpose [107].

Distance-based methods, for example, were useful to identify approximately 77% of the 528 species of Mexican cacti using sequences of the plastid gene *matK* (“matK barcode”) [108]. This strategy may have many practical applications, although its use is taxonomically limited due to challenges that preclude the adoption of a universal barcode strategy in plants [109]. A limiting factor, for instance, is the low genetic variability found in most plastid DNA sequences at the shallowest taxonomic level, leading to the need to combine several sequence markers. Moreover, another major issue is the lineage-specific variation as a consequence of heterogeneity in the evolutionary rate among plant lineages, such as those found in cactus [42], which limits a universal application of the same marker across broad taxonomic spectra.

The integrative framework assessing both molecular and phenotypic/ecological traits has been recently adopted to shed light on taxonomically species-level puzzling groups (e.g., [110,111,112,113]). Although this is not always explicit, the underlying species concept in most of these approaches is the practical phylogenetic concept [114,115] or the genotypic cluster concept [116]. For example, Aquino et al. [117] combined molecular (four cpDNA regions) and morphological data (quantitative and qualitative traits) to delimit species from the North American genus *Epithelantha* (subfamily Cactoideae, tribe Cacteae). In addition to performing species recognition, the authors were able to propose new species and a taxonomic key for species diagnosis in this genus. More recently, the same research group used environmental variables and soil characteristics to delimit the *Epithelantha* species [84]. In another study, Alvarado-Sizzo et al. [118] used congruent results obtained from genetic structure, inferred using SSR markers, ecological niche modeling, and morphological data to delimit four species of the *Stenocereus griseus* species complex (subfamily Cactoideae, tribe Cacteae).

Apart from the formal description, genetic data may also be useful to test alternative hypotheses in taxonomic unstable groups. For example, the *Pilosocereus aurisetus* group (Subfamily Cactoideae, Tribe Cereeae) comprises seven recognized species [4] but with a history of repeated taxonomic re-evaluations [119]. Khan et al. [48] used a set of molecular data (SSR and cpDNA regions) and a population structure approach to test taxonomic hypotheses regarding *Pilosocereus jauruensis*, a species exhibiting a sky-island distribution. The authors found populations of *P. jauruensis* showing species-level genetic divergence and geographically coincident with the occurrence of the heterotypic synonymous *P. densivillovus* [120]. Rather than resuscitating the obsolete name *P. densivillovus*, Khan et al. [48] highlighted the level of evolutionary independence of these *P. jauruensis* populations, which should be treated as independent units for conservation purposes. Recently, these results from Khan et al. [48] were used to support the proposition of a new subspecies, *P. jauruensis* subsp. *cincinnopetalus* [121].

The treatment given by Khan et al. [48] for their results is consistent with the General Lineage Concept of Species (GLCS), [122]. Briefly, in this concept, the incipient species are evolving metapopulation lineages in which the species’ attributes (e.g., phenotypic divergence; monophyly, reproductive isolation) are complementary stages of a process of continuous divergence and not a mandatory characteristic as in the traditional species concepts. The GLCS concept embraces the bridge between microevolutionary processes generating branching patterns, the core of Darwinism theory, and, more specifically, of the phylogeography discipline [123]. In this sense, the multispecies coalescent model (MSC) is a candidate approach to consistently delimit species according to the GLCS by the statistical modeling of the relationship between the gene trees and the lineage history [107,124]. There are several MSC methods available, including those based on full likelihood (e.g., GMYC [125]), Bayesian posterior probabilities (BPP) (e.g., [126]), and supervised [127] and unsupervised machine learning approaches [128].

In our literature survey, we found only one paper using MSC for species delimitation in a cactus taxon. Perez et al. [107] introduced a new framework coupling the MSC with deep learning algorithms (convolutional neural networks) to test alternative species delimitation hypotheses within the *P. aurisetus* species complex. They were able to select a taxonomic ‘splitter’ hypothesis circumscribing five species instead of the single one currently recognized. The *P. aurisetus* species complex shows characteristics that impose many additional challenges to species delimitation, such as recent divergence, a highly fragmented sky-island distribution, and several instances of local morphological differentiation. These characteristics are shared with many other cactus systems, suggesting that there is an avenue to be explored on species delimitation of cactus using coalescent theory. We stress that the methods for species delimitation are additional tools to assist taxonomists rather than operational criteria for species diagnosis. They surely may help in the identification of candidate lineages as a “species hypothesis” for the evaluation of specialists.

### 4.5. Hybridization

Natural hybridization plays an important role in plant evolution [129,130,131]. Introgressive hybridization, reinforcement, local extinction, or hybrid speciation are the most relevant outcomes of hybridization, but interspecific gene flow may lead to a variety of results depending on the level of divergence between species, geographical context, and the balance with selective forces [132]. The role of hybridization in cactus evolution has long been highlighted [10,13,15,27,28,133,134]. Registers of interspecific and intergeneric cactis hybrids are common in both nature and cultivation. The constancy in basic chromosome number and the presence of self-incompatible flowers are factors that would improve the chance for hybridization in many cacti [28], although the occurrence of hybridization seems to be more frequent in some clades in the family (e.g., genus *Opuntia*). Molecular markers are valuable allies to study the frequency and extension of hybridization [25]. Moreover, many statistical methods are available to investigate gene flow, depending on the depth of hybridization that one aims to detect [132]. For instance, these methods may involve the detection of genetic admixtures and F1 hybrids (e.g., *STRUCTURE* [135]; *NEWHYBRIDS* [136]), symmetric and asymmetric gene flow (e.g., *MIGRATE* [137]; *G-PhoCS* [138]), introgressive hybridization (e.g., *INTROGRESS* [139]), and gene tree inconsistencies (e.g., *SNaQ* [140]). Despite this, based on our survey criteria and categorization, we found relatively few studies exploring hybridization with molecular markers (Figure 4), most of which were related to our population genetics category (nine studies) followed by phylogenetics (five studies).

The genetic evidence for hybridization in the cactus family was generated using different approaches and molecular markers, such as analysis of RAPD banding pattern data [10,141], levels of admixture in codominant markers (allozymes [142]; SSR [143]) and biallelic SNPs generated by RAD-Seq [27] as well as observation of reticulations in phylogenies (e.g., [13,144]). Overall, the molecular data corroborate the occurrence of interspecific hybrids in different genera, such as *Opuntia* [10,141], *Sclerocactus* [145], and *Melocactus* [27]. Furthermore, molecular markers have assisted studies on intergeneric hybrids in the cactus family, such as *Consolea* × *Opuntia* hybrids in the subfamily Opuntioideae [13] and *Espostoa* × *Haageocereus* in the subfamily Cactoideae [28].

Moreover, molecular markers have been useful to test taxon-specific hypotheses on species boundaries and hybridization (e.g., [143,144,146]). Interestingly, independent results have shown the maintenance of species genetic cohesion even when facing some level of genetic admixture [27,145]. For example, Khan et al. [27] investigated four hybrid zones hosting *Melocactus concinnus* and four congeneric species (*Melocactus ernestii*, *Melocactus glaucescens*, *Melocactus paucispinus*, and *Melocactus zehntneri*). Briefly, the authors found intense hybridization in sympatric areas (with a bi and tri-species admixture) but with a low amount of introgression (2–5%), indicating that the studied species present weak pre-mating but strong post-mating reproductive isolation. These results highlight the importance of hybridization and natural selection in the maintenance of the genetic integrity of the *Melocactus* species.

The major role of hybridization in cactus speciation is recurrently explored in genera with high amounts of polyploids [13,14,28]. This is the case of the genus *Opuntia* [13,144], which presents approximately 60% of polyploid species [147]. Recently, using an integrative approach with phylogenetic, cytogenetic, and taxonomic datasets, Köhler et al. [14] showed that the octoploid (2n = 88) accessions of *Opuntia* aff. *ficus-indica* found in Santa Fe, Argentina likely presents a putative hybrid origin between the native *O. rioplatensis* and the introduced *O.* aff. *ficus-indica*. This hybrid form was described as *O. x cristalensis* [14].

Some hybridization studies in our survey have been conducted using SSR markers amplified with heterologous primers. Arakaki et al. [28] used plastid DNA and a set of nuclear SSR markers, formerly described for *Haageocereus* [148], to test hypotheses of hybridization involving species of *Espostoa* and *Haageocereus*. The SSR data showed evidence that hybrids between *Espostoa* and *Haageocereus* are viable, and that hybridization has gone beyond the F1 generation, suggesting the presence of hybrid swarms in some of the studied populations. However, although SSR data are useful to study hybridization, some statistical frameworks demand larger datasets.

Here, we recovered only one paper using genome-wide markers to explore hybrid zones [27]. It is worth mentioning that the use of RAD-Seq markers by Khan et al. [27] greatly improves the resolution of the genetic architecture of hybridization in the genus *Melocactus* in comparison with previous studies based on traditional markers or phenotypes (e.g., [142]). Undoubtedly, the study of hybridization in many Cactaceae taxa will be more accessible by the use of new sequencing technologies. The possibility of generating genomic datasets will allow the investigation of genetic variation across the entire genome, providing deep insights into the hybridization process in Cactaceae [132,149]. Specifically, for the cactus family, we seek to understand many open questions, such as: How permeable to gene flow are species? What is the importance of hybrid speciation for the group? What is the role of reinforcement in improving premating reproductive isolation?

### 4.6. Genome Assemblies

The studies of functional genomics within Cactaceae evaluated here (excluding studies restricted to structural or genome size estimation from flow cytometry) are clearly associated with the advance and availability of NGS methodologies in the past decade. The species’ samplings in these studies were focused on a few tribes, such as Phylocacteae, Cereeae, Cacteae, Cylindropuntiae, and Rhipsalideae (Table S3). The amount of nuclear genomic data is still underexplored in the family and focused on North American cacti diversity. Currently, we recovered only three papers describing genomic sequencing and assembly [15,30,150]. From them, we found a chromosome-scale genome assembly of *Hylocereus undatus*, a draft of the whole genome of *Carnegiae gigantea*, four low-coverage genomes of species from North American cacti (*Pachycereu springlei*, *Lophocereus schottii*, *Stenocereus thurberi*, and *Pereskia humboldtii*), and a low-coverage genome from a South American cactus species (*Cereus fernambucensis*). The number of plastomes (ten studies) and transcriptomes (six studies) available in public repositories has been increasing in the last few years (from 2015 to August 2021). This is probably due to the small size of the plastome organelle and gene length, lower need for bioinformatics skills, and lower hardware/software requirements applied for both plastome and transcriptome assembly approaches. This kind of information is of paramount importance, providing genomic resources and background for future studies of cacti lineages, including phylogenetics and phylogeographic studies.

The RNA-Seq method has been used in the family for different purposes (Table S3). For instance, Walker et al. [151] and Wang et al. [152] used this kind of dataset for phylogenomic and evolutionary studies, while other authors tested differential gene expression in cactus species under distinct ecological stress, drought event conditions, RNase-based self-incompatibility, gene expression in root development, and genes under positive selection into specific clades [30,153,154,155,156,157,158] (Table S3). Thus, the application of transcriptome methodologies to study ecological and environmental stresses may provide new evidence to understand cacti adaptations [30,159].

It is worth noting that several of these papers are from corresponding authors located in the USA or China (Table S3), the latter with non-occurrence of native cacti species. This observation suggests that the availability of NGS technology with a low cost, transcriptome/genome assembly expertise, and lack of functional genomic information within the Cactaceae family seems to be attractive for different research teams around the world. On the other hand, it is widely known that the use of genomic technologies is biased around the world, especially concerning inequalities in countries’ expenditures for research and development, lack of skilled personnel, and low quality/quantity of outsourcing services [160]. Regarding the costs, an interesting review published by McKain et al. [161] reports the approximate values for distinctive genomic strategies commonly used in plant studies.

Although genomics technology has advanced in the last decade, as well as the number of plant genomes available, the number of Cactaceae nuclear genomes does not follow the same pattern. Some methodological challenges might be associated with this observation, such as extraction of high-quality DNA/RNA solutions or genome assembly troubles associated with the level of ploidy, heterozygosity, and high repetitive sequence content, which seems common in Cactaceae [30]. Poor DNA/RNA quality and purity are problems commonly observed in succulent plants since they are rich in mucilage and secondary metabolites [162]. Distinct protocols are currently available for succulent plants, combining the use of commercial kits or the adaptation of existing ones [163]. Actually, in our survey, we observed the use of distinct protocols and distinctive tissues for nucleic acid extractions. Thus, it is important to test different methods and standardize them for particular taxon/tissue/laboratory conditions.

Challenges faced during genome assembly are most related to the use of bioinformatic knowledge, including software and hardware characteristics, to solve biological issues [164]. These features cause ambiguous mapping of sequences, promoting misassembled false genomes and erroneous arrangements [165,166]. The unanchored assembly sequences may create allelic variations that do not necessarily exist, which may be the cause of most of the problems [167]. There are no easy answers for these issues. A good genome assembly methodology practice requires the application of a distinct software assembler and the evaluation of the datasets to be merged into a final genome [168]. An additional challenge for plant genomics is provided for the growing evidence-based on comparative genomes suggesting large structural differences and presence–absence variances of gene content even between individuals of the same species [169]. This observation culminates in the concept of “pan-genomes”, associated with the investigation of multiple individuals to have a reliable reference genome [170], an issue relatively far from the current reality of cactus genomics but which will need to be addressed in the future. New sequencing methods/platforms and bioinformatic algorithms are still in development and in continuous improvement, which may overcome many of these challenges in the near future

## 5. Concluding Remarks and Future Directions

We report considerable growth in publications associated with evolutionary genetics (Figure 1a) and genome assemblies (Table S3) after the 2000s. These studies have added substantial knowledge on many issues. Despite this, many biases and challenges still remain in our field. For example: How can the challenges involved in new sequencing technologies be overcome? How can large datasets and phenotypic and environmental traits be better integrated and analyzed? How can the taxonomic (Figure 2) and geographical (Figure 3) biases observed in our survey be mitigated?

We believe that many collaborative efforts at different levels should be made with regards to these challenges. First, the results based on new markers have been promoting fundamental advances in both the ability to generate informative data and to reach theoretical advances in evolutionary genetics. Even though PCR-based markers remain informative for cacti research, the application of new methodologies seems to be appropriate to improve the statistical power in the estimation of evolutionary parameters, such as by using coalescent-based methods. Restriction digest-based methods (RAD-Seq and related technologies) are among the candidates to obtain a genome-wide set of loci shared in a sample of individuals [161]. As RAD-Seq requires no prior genomic resources whatsoever, it has become the most widely used genomic approach for high-throughput SNP genotyping in ecological and evolutionary studies of non-model organisms [171] and a possibility of choice for cactus researchers for both phylogenetics [62] and population-level analyses [27]. Reference-guided assemblies to process genome skimming data have also been successfully applied to resolve phylogenies in cactus species (e.g., [44]). Moreover, the recent description of two panels of orthologous genes specific for the cactus family [172,173] will certainly encourage researchers to generate multi-locus data in the short term. The advantage of such panels is better reproducibility and that they allow a better comparison of different cacti groups and plants.

The challenges to broadly implement NGS technologies are not restricted to technical issues (high-quality DNA/RNA, assemblies, etc.) but also involve other dimensions, such as funding and personal skills. For example, most cactus species naturally grow in developing countries with heterogenous challenges to research and development (R&D). Indeed, in low-income countries the investments in R&D are in general lower than 1% of gross domestic product (GPD) while this percentage is usually higher than 2% in nations with a global leadership in science [174]. Thus, despite the complexity and many variables of these issues, the cactologist community should be integrated and engaged in pushing governments to find solutions to at least a fraction of these challenges, since many species are under threat [93,94]. Fostering international collaborations may contribute to the transfer of technology and knowledge among researchers of different countries and regions around the globe. For example, the Earth Bio Genome Project (EBP) is an ambitious project aiming to characterize the genomes of all of Earth’s eukaryotic biodiversity over a period of 10 years, involving a network of researchers from all continents [175]. Certainly, this type of collaborative project should be encouraged in the microcosmos of cactus research through partnerships between researchers (or funding agencies) of high-, middle-, and low-income countries. In 2021, during the COVID-19 pandemic outbreak, the International virtual mini-symposium “Cactaceae: Phylogenetics, Evolution and Conservation in the Genomic Era” was realized [176]. This event put together an international community of experts allowing a holistic view of the research programs of many research groups. We are confident that this event might be a landmark toward the establishment of an international network of cactus research focused on evolutionary genetics.

International collaborations seem to also be necessary to obtain permits and improve sampling in a few explored areas, with a high diversity of species and endemism, as we have shown substantial taxonomic and geographic biases (Figure 2 and Figure 3). Several clades were poorly explored, including groups that are not recovered in any work, such as the genera *Corryocactus*, *Calymmanthium*, and *Frailea*. The bias in the number of studied species and domains seems to be associated with the number of publications in the countries/regions where these species occur or are distributed. The formation of human resources in such areas would also be imperative to mitigate the historical biases throughout a new generation of researchers interested in cactus diversity.

Finally, to better integrate different source data in predictive analysis [97,98], initiatives toward the establishment of common databases and data availability are necessary. Moreover, it is also important to improve the incentive in the generation of phenotypic, environmental, and life history trait datasets, which are still lacking for most cactus clades. In this sense, collaborative efforts to integrate geneticists, ecologists, botanists, and evolutionary biologists seem to be imperative to understand different dimensions of cactus diversity. Cactus are relevant models to understand evolutionary principles and biogeographic processes, and their study has inspired generations of researchers over time. Mitigating the taxonomic/geographic biases and the implementation of NGS technologies associated with multi-locus and integrative statistical frameworks seems to be necessary steps to better understand this emblematic group of plants.

## Data Availability

No new data were created or analyzed in this study. Data sharing is not applicable to this article.

## Supplementary Materials

The supporting information can be downloaded at: https://www.mdpi.com/article/10.3390/genes13030452/s1. Figure S1: Flow diagrams of the systematic review performed in this review; Figure S2: Distribution of cactus studies by the first author’s country; Figure S3: Number of studies using each type of molecular marker; Figure S4: The main biogeographic theories addressed in the evolutionary studies of Cactaceae; Table S1: Summary of reviewed studies on evolutionary genetics of cacti classified as the main topics “Populations genetics” and “Phylogeography”; Table S2: Summary of reviewed studies on evolutionary genetics of cacti classified as the main topic “Phylogeny”; Table S3: Summary of reviewed studies on evolutionary genetics of cacti classified as the main topics “Genome/Transcriptome”; Table S4: Frequently referenced theories in biogeographic studies of cacti and the number of studies associated with each one.
