# Supplementary material for: Evolutionary Genetics of Cacti: Research Biases, Advances and Prospects
Source: Genes (Basel). 2022 Mar 1;13(3):452. doi: 10.3390/genes13030452 (PMC8952820; doi:10.3390/genes13030452)
Supplement: Supplementary file 1 [file genes-13-00452-s001.zip › genes-1590307-supplementary/SM_Figures_R1.pdf]

SUPLEMENTARY MATERIAL

Evolutionary Genetics of Cacti: research biases, advances and prospects

By Fernando F. Franco[1,#,*], Danilo T. Amaral[1,2,#], Isabel A. S. Bonatelli[3,#], Monique Romeiro-Brito[1,#] , Milena C. Telhe[1,#] and Evandro M. Moraes[1,#]

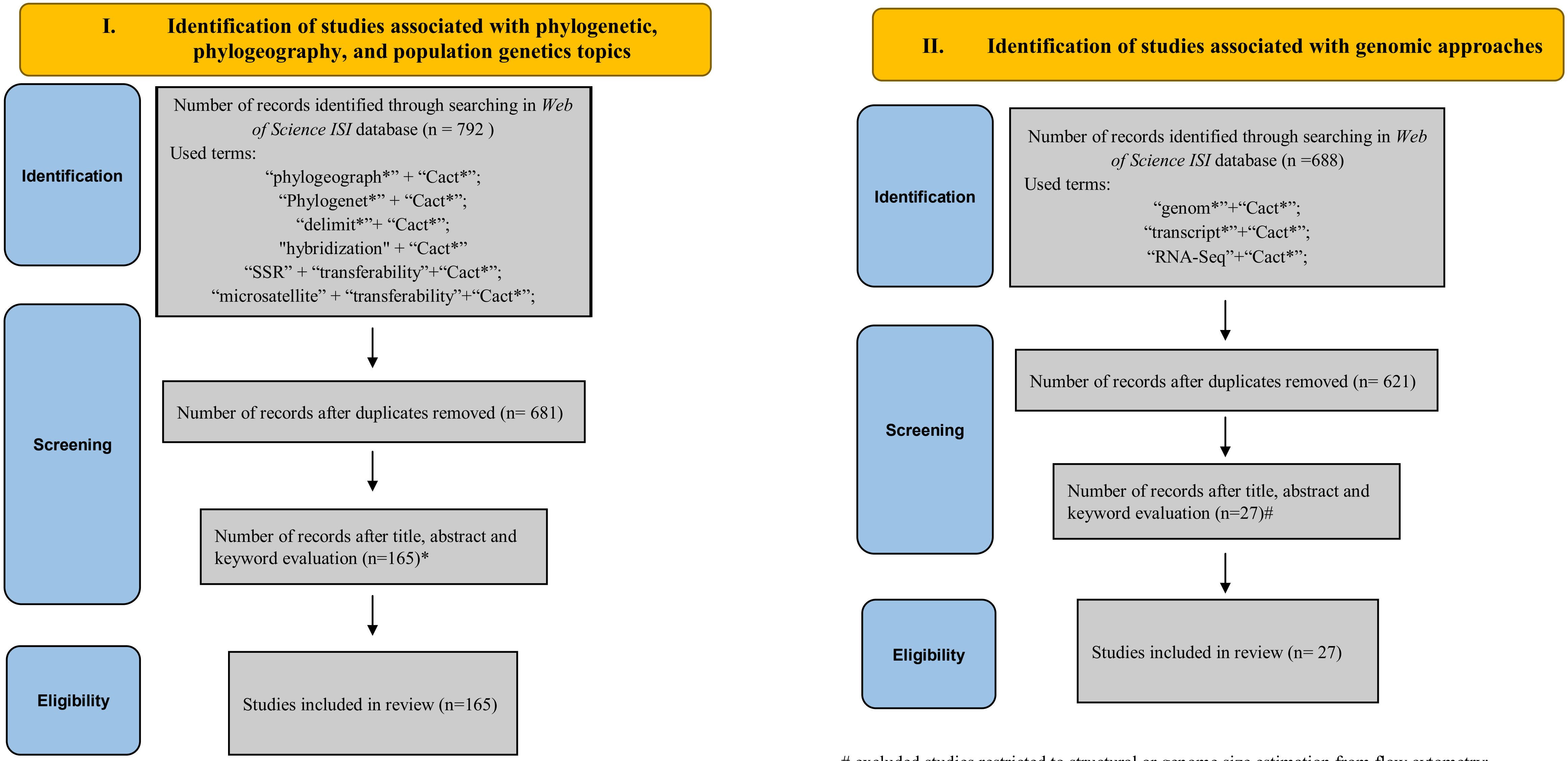

* excluded allies and cultivate species; associated species (e.g., cactophylic drosophila)

# excluded studies restricted to structural or genome size estimation from flow cytometry; associated species (e.g., cactophylic drosophila)

Figure S1. Flow diagrams of the systematic review performed in this review.

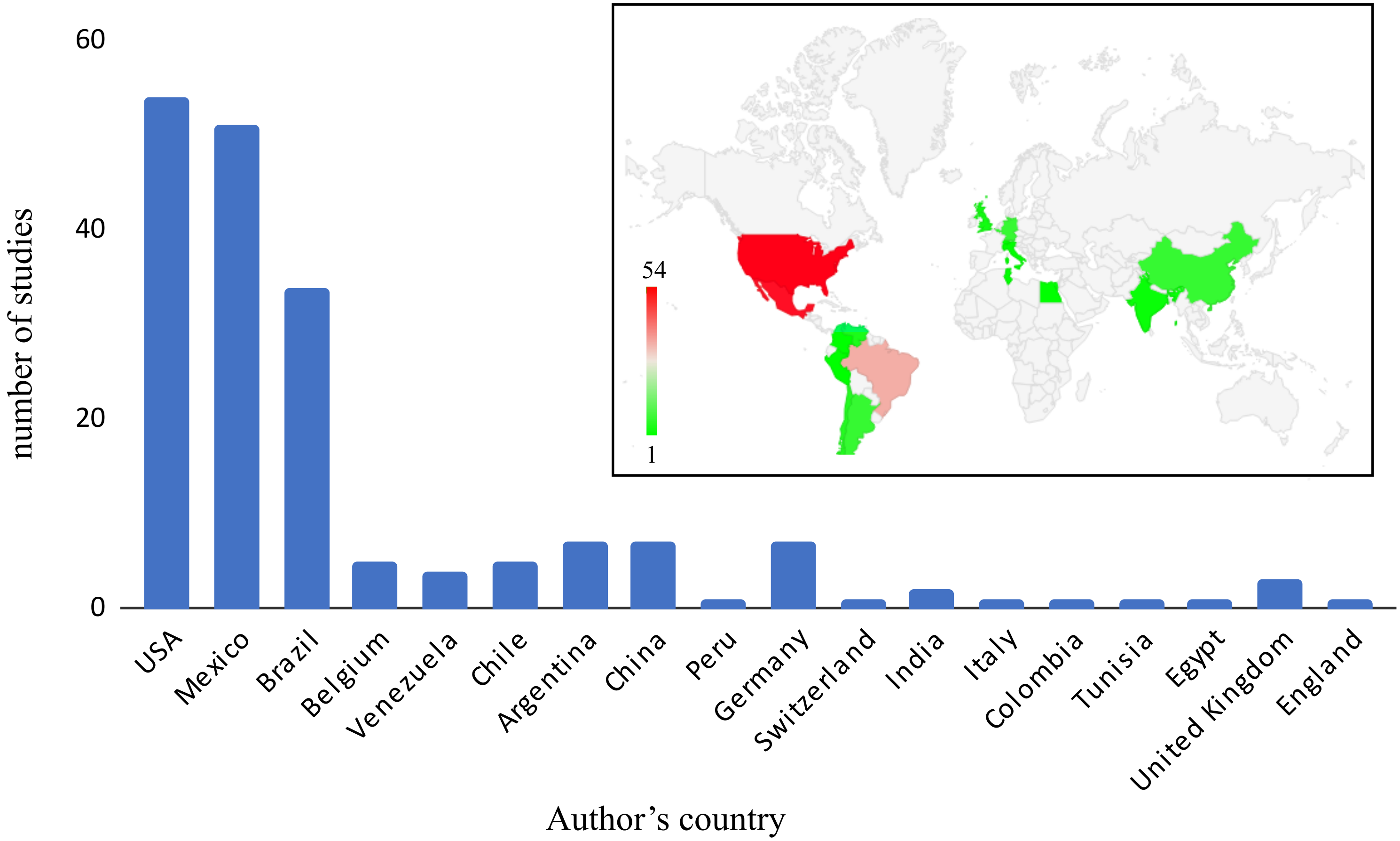

Figure S2. Distribution of cactus studies by the first author's country.

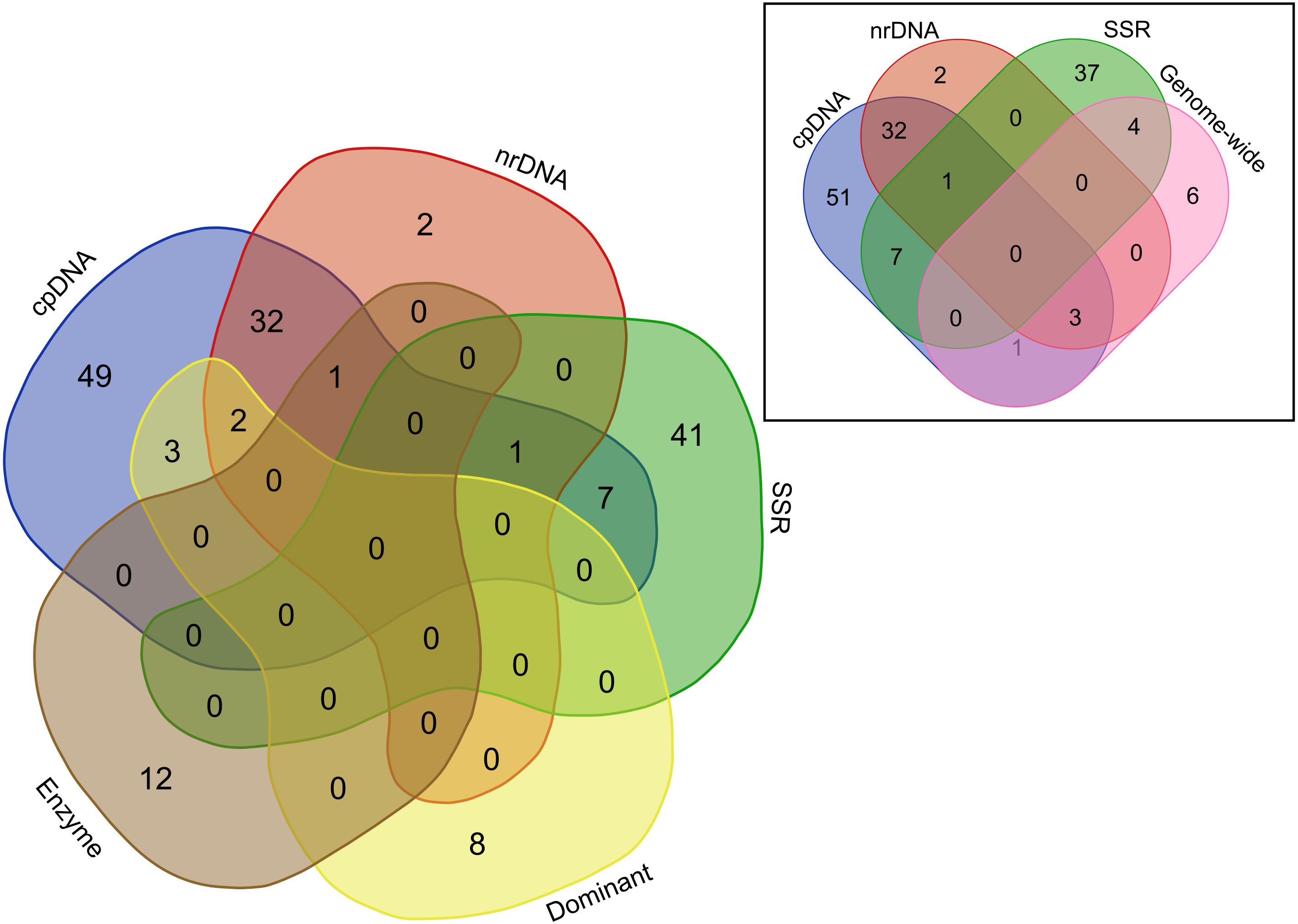

Figure S3. Number of studies using each type of molecular marker. The insert box shows the number of studies according to the most widely used markers.

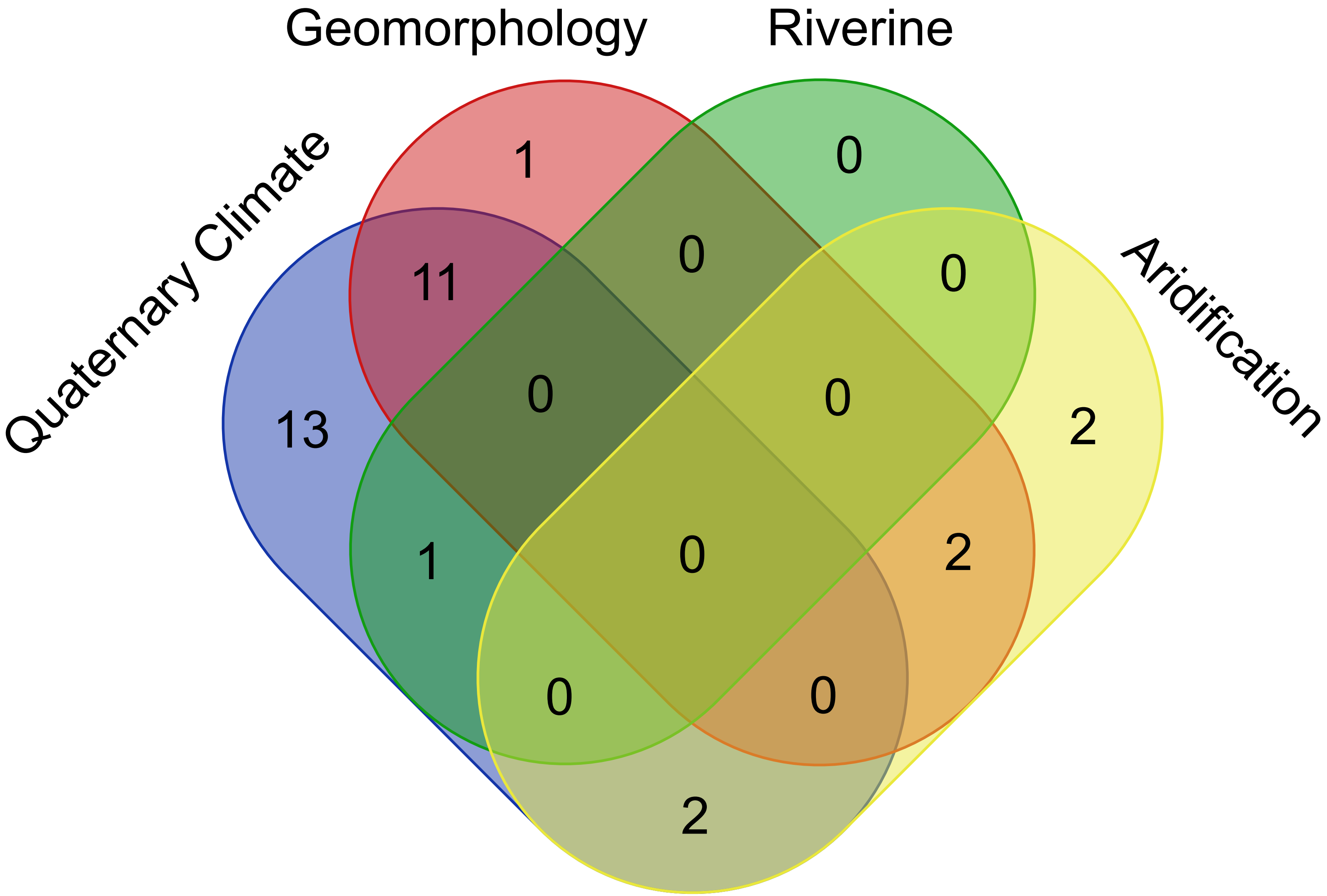

Figure S4. The main biogeographic theories addressed in the evolutionary studies of Cactaceae. Numbers indicate the number of studies.
